# Home Health Care (HHC) Managers Perceptions About Challenges and Obstacles that Hinder HHC Services in Jordan

**DOI:** 10.5539/gjhs.v7n4p121

**Published:** 2014-12-31

**Authors:** Musa T. Ajlouni, Hania Dawani, Salah M. Diab

**Affiliations:** 1 Philadelphia University, Department of Hospital, Amman, Jordan; 2 Jordanian Nursing Council, Amman; 3 Business Administration Departments, Applied Science University, Amman

**Keywords:** home health care services, managers of home health care agencies, home nursing, challenges and obstacles, Jordan

## Abstract

Home care aims at supporting people with various degrees of dependency to remain at home rather than use residential, long-term, or institutional-based nursing care. Demographic, epidemiological, social, and cultural trends in Jordan as in other countries are changing the traditional patterns of care with growing emphasis on home care. The purpose of this study is to highlight the most common challenges related to home health care (HHC) services in Jordan as perceived by the managers of HHC agencies. Methods: a descriptive qualitative design that depends on focus group discussions has been used to collect data from a sample of 18 managers who met the selection criteria and who are willing to participate, the study found that, the main challenges of HHC services as perceived by managers were: shortage of female staff, lack of governance and regulation, poor management, unethical practices, lack of referral systems, and low accessibility of the poor and less privileged as HHC services are not included in health insurance schemes, it concludes also that the home health care industry in Jordan is facing many challenges and problems that may have negative effects on the effectiveness, efficiency, equity and quality of services and should be addressed by health policy makers.

## 1. Introduction

Home care aims at supporting people with various degrees of dependency to remain at home rather than use residential, long-term, or institutional-based nursing care. Home care providers render services in the clients’ own home. These services may include combined professional health care services and life assistance services. Professional home health services could include medical and/or psychological assessment and intervention: wound care, medication teaching, pain management, disease education and management physical therapy speech therapy and occupational therapy (Shepperd, 1998).

Demographic, epidemiological, social, and cultural trends in Jordan as in other countries are changing the traditional patterns of care, which means that all the changing conditions in the region are the same such as, improving technology, increased the educational levels, increasing in population rate, which is around 2.5% (according to Jordanian ministry of health un-published report). The next decades will see increasing rates of care-dependent older people and non-communicable diseases as the leading cause of chronic illness and disability. The break-up of the traditional large family group and urbanization will also lead to gaps in the care of older or disabled family members (Ajlouni, 2011).

According to the data provided by the Ministry of health, Jordan has a total of fortysix (46) private home care agencies. Thirty-six of these are located in Amman, four in Irbid, three in Zarqa, two in Balqa, and one (1) in Mafraq. Other than the location of these services, nothing is detailed in relation to the structure, types or population served. There are no legislation, policies or guidelines that govern home health care services in Jordan, therefore these services are neither regulated nor monitored and their accountability to the users of the services is not defined.

Thus, there is an urgent need to explore the current status of the home health care sector in Jordan and explore the conditions that facilitate or hinder provision of HHC as perceived by managers of HHC.

## 2. Protection of Participants Human Rights

Prior to the sessions, participants were asked to sign a consent form. The sessions were audio taped and participants agreed on this. Participants’ rights to confidentiality, privacy and safety were protected throughout the study. Files were kept in locked cabinets at the HNC. All projects’ electronic versions were kept in the primary investigator’s computer only. An approval from the Academic Research Committee at Philadelphia University was obtained at the beginning of the research project.

## 3. Objectives

The purpose of this study is to highlight the most common challenges related to home health care (HHC) in Jordan as perceived by managers of HHC. The specific objectives are:

1) To assess managers’ perception of the current situation of home health care services in Jordan.
2) To assess managers’ perception of the conditions which facilitate or hinder provision of home health care services in Jordan.

## 4. Methodology

### 4.1 Study Design

This is a descriptive qualitative study that depends mainly on focus group discussions for gaining insight into participants’ perception and experience related to the phenomenon under study.

### 4.2 The Sample Study

All of the 46 home health care agencies in Jordan are targeted. Eligibility criteria stated that the manager should have a management/administrative position in the agency, and a minimum of 6-month experience in the management/administration of the agency with proper knowledge of the agency business.

Of the thirty-five mangers who met the selection criteria, only 18 managers were willing to participate in the focus group discussions. Participants were representing HHC agencies located at Amman and Zarka cities. The participants were randomly allocated into two subgroups (9 persons per group).

### 4.3 Data Collection (Focus Group Discussions)

Each subgroup had a focus group session of 90 minutes. All focus group sessions were conducted at the High Nursing Council (HNC) office in Amman. Issues raised during the focus group discussions cover many aspects of home health care services as users health conditions, staffing, staff qualifications, referrals, payment schemes, pattern of utilization and barriers to utilization, quality and ethical issues, in addition to challenges facing Jordan HHC services as perceived by participants and suggestions to face these challenges (Annex 1). The focus group discussions were conducted by the researchers.

### 4.4 Managers’ Characteristics (General Characteristics of the Study Sample Individuals)

Table (1) indicates that (78.9%) of managers (n=15) are males and 21.1% (n=4) are females. The age categories shows almost equal distribution as 26.3% are at age less than 30 years and 36.9% are at age 30-40 years, and 33.1% are above the age of 40 years. Only one manager (5.3%) is none Jordanian while the others are Jordanians. Most of the managers have bachelor and master degrees (42.15% and 26.35% respectively), while 26.3% of them have less than bachelor degree and only one manager has a PhD degree. The majority of the managers also are nurses (73.7% (n=14) while there is one physician (5.3%) and the others (21.0%, n=4) are from different health specialties. Regarding experience in home health services business, results show that 50% of the managers (n=9) are (5) years or less in business.

**Table 1:** Demographic characteristics of managers of Home Health Care Agencies

Variable		N (18)	PERCENT
**Gender**	Male	14	77.8
	Female	4	22.2

**Age (years)**	< 30	5	27.8
	30 – 39	6	33.3
	40 – 49	4	22.2
	50 - 59	3	16.7

**Qualification**	High school	2	11.1
	Diploma	3	16.7
	BSc.	7	38.9
	Master	5	27.8
	PhD	1	5.5

**Job**	Physician	1	5.5
	Nurse	13	72.3
	Physiotherapist	3	16.7
	Laboratory technician	1	5.5

**Years in business**	1 – 2	5	27.8
	3 - 5	4	22.2
	6 – 8	3	16.7
	9 – 15	3	16.7
	16 – 20	2	11.1
	> 20	1	5.6

### 4.5 General Characteristics of HHC Agencies

Table 2 shows that the majority of home health care agencies (HHCAs) are located and providing their services in the capital city of Amman (88.8%). Although the locations of agencies are limited to Amman and Zearqa, the agencies reported that they provide services to other cities such as Irbid and Jerash. Regarding type of services, all agencies (100%) reported that they provide nursing care services. In addition to nursing care, most of the agencies provide services related to medical equipment rental and physiotherapy (77.8% and 66.7% respectively). (44.4%) of agencies reported that they have training programs to their employees.

**Table 2:** Characteristics of Home Health Care Agencies

Variable		N(18)	PERCENT
**Agency location**	Amman	16	88.8
	Zarqa	2	11.2

**Target area of services**	Amman	17	94.4
	Irbid	5	27.7
	Zarqa	9	50.0
	Aqaba	1	5.6
	Karak	1	5.6
	Jerash	2	11.1
	Salt	1	5.6

**Type of services**	Home Nursing Care	18	100
	Physiotherapy	12	66.7
	Occupational therapy	5	27.7
	Medical equipment and supplies rental	13	77.8
	Babysitting	1	5.6
	Transportation to health care setting	1	5.6
	Geriatric care	1	5.6

**Training**	Offer training programs to staff	8	44.4

**Nationality of Patients**	Jordanians only	15	83.3
	Jordanians and Non-Jordanians	3	16.7

## 5. Focus Groups Discussions

### 5.1 Staffing Discussions

While nurses (registered and associated) are the main employees working at HHCAs, some agencies have also reported that they employ other professionals as vocational nurses, physiotherapists, occupational therapists, and physicians. More than 50% of the agencies do employ full time staff.

The shortage of female nurses poses problems to most of the agencies. The following reflects this problem:” Shortage of female nurses causes problems for us, not only in terms of serving patients, it also results in recruiting workers without proper qualifications and good experience, which puts us in trouble related to quality of service provided to our clients. Some female nurses are not allowed by their families to work at homes, especially during evening and night shifts. I am trying to recruit female nurses from the Philippine; at least, I will not be under pressure of staff shortage”. Hiring unqualified staff was another concern as reflected in this quotation: “Most of the agencies hire unqualified workers; this undermines our work and lowers the trust in our services.”

### 5.2 Referral System

Most of the participants in the focus group reported concern over the lack of referral systems from hospitals, despite individualized informal referrals by hospital staff based on friendship or financial returns. Two participants, however, reported receiving referrals from physicians and from hospitals on personal relations basis. One agency actually has contracts with some hospitals.

The participants reported the lack of referral system as expressed in the following:

“We tried to contact hospitals and establish a referral system with them. The supervisors, however refused because they choose to please their nurses… may be they benefit too!”

“If our services get to be known to users, hospital admissions will go down.”

“Hospitals will continue to block our work, they are afraid of the threat of home health care on their profit.”

Reliable services as evident in two accounts of the participants, succeeded in establishing referral agreements with hospitals. These two participants had well-established services, and adequate numbers of full time registered nurses. The following expressed their view on the subject:

“Reputable agencies who deliver quality develop trusting relationships with physicians who consistently refer patients. I have a good relationship with doctors, mostly specialists, and with nurse administrators. You said (addressing other participants) that you do not receive hospital referrals, but my agency does, we have contracts with hospitals.”

### 5.3 Governance and Regulation

The lack of governance of HHC services and the need for regulation was a theme that was highlighted by all the participants. They perceived lack of support and did not know who to communicate with, and how, if and when they had problems or grievances to report. They felt that home health care industry is not organized and has loose boundaries, and gaps that do not define the work of HHC providers especially those who are free-lance and work on their own.

Half of the participants yearned for The Ministry of Health involvement as expressed in the following statements:

“Though the Ministry of Health (MOH) should regulate and monitor all health services in the country as mandated by law, it is almost absent regarding HHC. We feel all alone fighting many issues that challenge our work. The families also do not know who to report to when they have problems.”

“The MOH gives us a piece of paper (license); it is obtained in one visit to the Ministry, I never saw them again.”

“During my work in the agency for the last 5 years, we were visited only once by MOH staff for monitoring objectives; they asked us about number of nurses and whether we sell medical equipment or not. (Implying that monitoring is not effective)”

“I do not know where to go if I have a problem.”

“Taxi drivers bring in customers… who should organize the work? Is this allowed or not allowed?”

The participants raised issues related to requirements and guidelines, which reflected their incomplete knowledge of the governing laws and bylaws. Participants however, knew that they were not allowed to sell medical equipment under the home care nursing licensure, but chose to do so as expressed in the following:

“We opt to sell and rent medical equipment… profit does not come from nursing care, we need to supplement our income through commission from rentals or income from sale of basic medical supplies, wheel chairs, beds and medical equipment as monitors, ventilators and sophisticated equipment necessary for specialized care.”

### 5.4 Management Issues

Almost all the participants perceived disorganization as one of the hindrances in their work, they felt that the lack of active governance was the main reason of this disorganization, and expressed the need for collaboration among each other to support one another and to set the rules for working together and for fair competition. The participants complained about the lack of information and reports that facilitate the planning and the management of their work.

Some of these concerns are supported by the following statements:

“Our work is not well planned or organized; it is so chaotic: locating patients, relating to other agencies, discontinuing services and discharge patients are all a mess.”

“Few agencies, if any, keep organized medical records for their patients or have written policies or procedures or job descriptions.”

“The nurses who work on their own do not report to anybody, who will support them if they face problems? Besides, they work without offices; they do not pay taxes as we do.”

### 5.5 Health Insurance and Affordability of Services

All participants reported that home health care is not covered by insurance and that most of service users pay out of pocket. This fact limits the services to those who can afford it and who are mostly residents of Western Amman. The Agencies registered to serve other governorates also serve in Amman because clients of areas other than Amman who can pay the fees are very few. Sometimes agencies accept patients from the public health sectors.

“Most, if not all, of our patients are rich or supported by rich families, since health insurance does not cover our services; our clients are those who can pay the fees.”

“Sometimes we discontinue our services to patients for financial reasons.”

It was clear that the payment schemes even within each agency varied according to the complexity and also the hours covered. The daily fees for a nurse as reported by the managers ranged between JD 30-35 per shift, while independent nurses charge JD 50.

The data revealed that the participants consistently compared the cost of hospitalization to that of home health care, and advocated for the coverage of this service by health insurance.

All participants support expanding health insurance benefits to include HHC as expressed by this quotation:

“We urge health insurers in the public and private sectors to cover the cost of HHC services, especially for patients with chronic diseases and disabilities, as this will minimize the cost of admission to hospitals, cover more patients and open the door wide to our industry.”

### 5.6 Ethical Issues

Most of the managers reported examples of unethical practices that include hiring of unqualified workers to care for patients with complex problems as evidenced in the following statements:

“We all know that many agencies send unqualified nurses to care for patients with serious problems….. They undermine our work and our reputation.”

Another concern that invited a heated discussion among the participants was the request for commission by colleagues, physicians and other workers, as evidenced and validated in the following accounts:

“Hospital staff asks for commission on patients referred (informally) to us. They ask for high commission. These hospital hustlers and nurse brokers are difficult to be controlled by their administration.”

“My relationship is with physicians, however, I never ignore the nurses, even when a case is transferred to me through a doctor I have to acknowledge the nurses, for some nurses the patient is considered as a commercial good…”

“Not only nurses ask for commission, housekeeping workers expect it too!”

“What are you talking about; I once had a doctor who called me, to tell me he has a case for me to sell!!”

“A physician saw me in the hospital, he stopped me saying ‘I have a patient for you, fifty- fifty!”

Few participants supported giving a percentage to the referring nurse for example:

“If he is your friend and he refers a patient to you, then you have to give him something, otherwise he will send the patient to someone who pays him.”

Managers reported that other agencies may entice and lure clients and persuade them to leave their services for a discounted rate. One participant bitterly reported the following:

“On the third day of admission of a bed ridden patient to our services, the manager of other agency, whom I know very well, called me laughingly and said ‘I stole your patient.”

Further concern expressed by the participants was related to families supporting unethical practices as follows:

“Families’ behavior, at times, is manipulative and dishonest. In trying to save money, families feed into dishonest practices.”

“Families need our nurses, but they do not want to pay the agency’s administrative fees, after we send them our nurses, they try to contract them independently!”

All participants claimed that the absence of governance and regulating bodies are responsible for most of these unethical practices as expressed by this statement:

“Name me an office to report to, and I am willing to report all bad and illegal activities. I guarantee you; we will clean up the mess in a month.”

## 6. Discussion the Study Results

The study found that (78.9%) of managers are males, 26.3% are at age less than 30 years and 36.9% are at age 30-40 years, and 33.1% are above the age of 40 years. Only one manager (5.3%) is none Jordanian while the others are Jordanians. 42.15% of the managers have bachelor, and 26.35 % have master degrees, 73.7% of the managers are nurses, 5.3% physician 21.0% different health specialties, 50% of the managers have (5) years or less experience in home health services business,.

(88.8%) of home health care agencies (HHCAs) are located and providing their services in Amman., all agencies (100%) reported that they provide nursing care services. 77.8% of the agencies provide services related to medical equipment, rental, and66.7% physiotherapy, (44.4%) of agencies reported that they have training programs to their employees.

Shortage of female nurses in Jordan is a common problem for all sectors and at all levels of care (Ajlouni, 2010). This problem is compounded by cultural norms of limiting their working hours to daytime and to contained work environment in hospitals. Working in unpredictable home environments of users is viewed as unacceptable and unsafe for females.

Shortage of female nurses especially in home care is not confined to Jordan; it is a worldwide problem. Ellenbecker and Cushman (2001) performed a study about the nurse shortage from a home care agency perspective. They found that certified home care agencies are challenged to meet an increasing demand for services in USA while faced with a predicted severe nursing shortage. Chenoweth et al. (2010) conducted a study to find factors that attract and retain nurses in aged and dementia care. They found that a family-friendly, learning environment that values and nurtures its nursing staff, in the same way as nurses are expected to value and care for their patients and residents, is critical in ensuring their retention in dementia and aged care.

Letvac (2001) stated that the different roles assigned to women in today’s society are burdensome, particularly for nurses who deal with the stress of managed care, downsizing, the nursing shortage, caring for increasingly ill patients, long and irregular hours, and daily crises. These issues should be addressed by homecare mangers to help female nurses cope with the rigors of the workplace.

Solutions to female nurse staffing problems in Jordan require careful study and strategic planning at the state and agency levels. Strategies are needed to motivate young women to enroll in nursing faculties to meet the shortage of female nurses like providing more scholarships and improving work conditions for female nurses. Also, managers of HHCAs should create a “Caregiver-friendly” practices such as creative, flexible scheduling; fair financial incentives and continuous training programs for attracting female nurses to home health care.

The difficulty in establishing referral agreements with the hospitals and with physicians can be related to the perceived capabilities of the agencies; most of the participating agencies did not have full time staff, they usually hire part-time staff according to patient load.

Appropriate and efficient provision of services at home requires more than skilled personnel; effective communication between the hospitals and home health care agencies is an often overlooked but essential tool for maintaining the patient’s health in the community. A method was developed in some hospitals in the USA to create home care orders that guides the physician through the order writing process, uploads data from the electronic medical record, and creates a legible, complete order set that can be faxed quickly to the agency (Siegler, 2007).

Castro et al. (1998) performed a study to assess the need for home health care referral screening for elderly patients after emergency department (ED) discharge. They concluded that “If home care referral screenings of elderly ED patients are performed and appropriate referrals are made before ED discharge, a seamless delivery system of health care is provided. A home care visit resulting from a referral may be all that is needed for the maintenance of a patient’s condition. To improve the quality and continuity of patient care, home care screening should be integrated into the routine discharge ED activities.”

Since proper home care has a significant impact on reducing hospital days, containing health costs and improving the quality and continuity of patient care as discussed before, hospitals and home health care agencies are encouraged to develop effective professional partnerships to refer patients from hospitals to agencies and vice versa.

The Health Professions and Institutions Licensing Directorate at the Ministry of Health (MOH) are the regulatory body of services rendered in the private sector including home health care services. This directorate holds a listing of licensed home health care agencies. However, its regulatory processes is limited to issuing of licensure to services according to a set criteria that requires that the applicant (owner-to be) of the agency is a registered nurse with five years of experience.

The lack of governance of home health care agencies in Jordan that operate in the absence of standards, guidelines and mechanisms for monitoring and following up, invites a whole array of legal and ethical misconduct, disorganization and a loose system that is not, and cannot be held accountable to service users or any other stakeholder.

The managers of HHCAs raised many important issues, which reflect poor management practices, and absence of basic governance principles. The results of this study raise an important issue of financial accessibility and equity regarding home health care services in Jordan. Poor and less advantaged patients can’t receive these services. Therefore, financial barriers should be addressed by health policy makers to make home health services in Jordan more equitable and accessible to all irrespective of their ability to pay.

Welfare states and wealthy countries either provide home health care services (HHCS) directly to patients through not-for profit state agencies or subsidy these services through social health insurance or social security programs. In England HHCS are provided by the state free of charge under the National Health Services (Klein, 1995).

In the USA these services are provided by for profit agencies and the fees are reimbursed by the government under the Medicaid and Medicare health insurance programs. There is no out-of-pocket deductible or co-payment for the beneficiary except for medical equipment items (Talaga, 2013).

Some government hospitals in Saudi Arabia have special programs for home health care. Abdul-Aziz University Hospital, as an example, provides home visits for patients with disability and chronic illness; including those with terminal illness. These services are provided free of charges for Saudi citizens. (KAUH - Home Health Care, 2011).

Concerns over ethical conduct focused on money related matters as commission, competition, luring cases, and families escaping administrative fees and contracting the nurses directly. Themes of regulation were also colored with financial concerns related to workers competing over the business that hindered money making for the agencies. Referral concerns were also issues related to the hospitals denying them the access to patients and to potential business shares. This clearly indicates that home health care industry in Jordan is not well organized and controlled, it also lacks basic standards and codes for professional practice.

Unethical practices in healthcare are a serious problem that face health care systems in all countries and not confined to home health care industry or specific country. Professor Bernard Lown (1999), Nobel Peace laureate and outstanding cardiologist, has described unethical practices in his book titled “The Lost Art of Healing” by saying: “We are sometimes baffled by the competing demands of apposite medical practices, personal biases, moral hazard (i.e. conflict of interest decision making) and the pervasive market-driven consumerism, so much so that we have subsumed our nobler instincts, and have lost our humane compassionate touch.”

Lauxen (2009) performed a qualitative ethnographic study to explore moral problems in the daily practice of nurses at home health care in Germany. There were four types of moral problems: “beneficence vs. autonomy”, “beneficence vs. justice”, “beneficence vs. loyalty” and “The good cannot be determined”. Some participants lack ethical competencies. Furthermore, appropriate support services for dealing with moral problems have to be designed.

Miller (1998) mentioned eleven unethical home care practices with emphasis on mental health care. They include: disregarding personal and medical privacy, using deceptive language violating traditional scientific ethics, practicing outside of a professional’s area of competence, creating and intensifying conflicts of interest, keeping secrets about financial conflicts of interest, violating informed consent procedures, using” fee splitting” or “kickbacks”, squandering money entrusted to their care, and disregarding information about harm to patients.

In USA, an inquiry by the Senate Finance Committee in 2011 (Martin, 2011) has found that the nation’s three largest home-health companies tailored the care they provided to Medicare patients to maximize their reimbursements and profits from the federal program. Strategies used at these companies included the designation of an “A-Team” tasked with developing programs to target the most profitable Medicare therapy treatment patterns and maximize Medicare reimbursements, and increasing the number of therapy visits to increase mix and trigger bonus payments.

However, to minimize unethical practices and moral hazards in home health practice in Jordan, it is recommended that a code of ethics should be developed and indorsed by all home health care agencies, also MOH and other health professional associations should activate their role in monitoring and controlling health care practices in the country including home care.

## 7. Conclusions and Recommendations

This study uncovers many challenges and problems that hinder the performance of home health care industry in Jordan and affect negatively on the effectiveness, efficiency and quality of services delivered to patients as: absence of professional guidelines practice protocols and quality indicators, lack of control and monitoring by health authorities, shortage of professional female nurses, lack of discharge planning and referral system from hospitals to HHCAs and vice versa, lack of national service fees schedule, lack of health insurance coverage, inadequate licensing and resource systems for caregivers and lack of training programs, shortage of written policies and procedures, lack of health information system including medical records keeping, prevalence of some unethical practices, poor governance and management practices and overemphasis and domination of profit making aspect on the expense of quality improvement.

To address these challenges the researchers recommend: development of partnerships, affiliation or even mergers among HHCAs; adopt professional guidelines, practice protocols and quality indicators for home health care services; controlling and monitoring performance of HHCAs by MOH; health insures schemes should be encouraged to include home health care services to their insurance package; HHCAs should develop written policies and procedures, health information system, medical records system, continuous training programs and proper management practices; development of a national service fees schedule for home health care services; hospitals and HHCAs are encouraged to develop effective professional partnerships and effective communication to refer patients from hospitals to agencies and vice versa; HHCAs should create a “Caregiver-friendly” practices such as creative, flexible scheduling, fair financial incentives and continuous training programs for attracting female nurses; and a code of ethics should be developed and indorsed by all home health care agencies to minimize unethical practices and moral hazards.

## Annex 1: Perception of Home Health Care Managers about Challenges related to HHC Services in Jordan: Focus Group Agenda

Site: Date: Time:

Participants (the attached annex about participants and their agencies should be completed by each participant).

**1. Introduction**

A focus group is a *qualitative* research method, used for gaining insight into participants’ perceptions the phenomenon under study (Challenges related to HHC Services in Jordan). A typical focus group consists of a facilitated group discussion, led by the focus group interviewer (facilitator), and based upon a series of open-ended questions. The participants are encouraged to engage in an informal discussion of two hours. The facilitator moderates the dialogue, according to a carefully designed plan, to ensure that no one or more participants’ views dominates the discussion. The opinions and views of the group must be summarized in a coherent and practical way.

**2. Objectives of the Tool**

The purpose of this tool is to highlight the most common challenges related to home health care (HHC) in Jordan as perceived by the participants and suggest policy directions to overcome these challenges. The specific objectives are:

- To assess managers’ perception of the current situation of home health care services in Jordan.
- To assess managers’ perception of the conditions which facilitate or hinder provision of home health care services in Jordan.
- To gather managers suggestions for further improvements of home healthcare services in Jordan.

3. Program

1) The facilitator welcomes the participants, introduces himself to them and asks everyone to introduce him/herself to the group.
2) The facilitator gives a 10 minute brief about the study, objectives and agenda of the meeting and methodology of discussions.

4. Session one

The participants are asked to give their feedback and opinion about the following HHC topics:

□ Staffing (categories, working hours, fees and salaries, main staffing issues)
□ Clients (main health problems, health insurance, hours of contacts)
□ Referral system (referrals from hospitals to HHC, referrals from HHC to hospitals).
□ Management (policies and procedures, records keeping, governance issues).
□ Quality of services: standards of care, professional monitoring).
□ Ethical issues: (common unethical practices, moral hazards).

5. Session two:

The participants are asked to discuss the following questions and agree on answers:

□ What are the main issues and concerns related to HHC in Jordan?
□ What are your suggestions to address these issues?
